# Image-based crosstalk analysis of cell–cell interactions during sprouting angiogenesis using blood-vessel-on-a-chip

**DOI:** 10.1186/s13287-022-03223-1

**Published:** 2022-12-27

**Authors:** Takanori Sano, Tadaaki Nakajima, Koharu Alicia Senda, Shizuka Nakano, Mizuho Yamato, Yukinori Ikeda, Hedele Zeng, Jun-ichi Kawabe, Yukiko T. Matsunaga

**Affiliations:** 1 Institute of Industrial Science, The University of Tokyo, 4-6-1 Komaba, Meguro-ku, Tokyo, 153-8505 Japan; 2 Department of Science, Yokohama City University, 22-2 Seto, Kanazawa-ku, Yokohama, Kanagawa 236-0027 Japan; 3 Hiroo Gakuen Junior and Senior High School, 5-1-14 Minami Azabu, Minato-ku, Tokyo, 106-0047 Japan; 4 Department of Biochemistry, Asahikawa Medical University, 2-1-1 Midorigaoka-higashi, Asahikawa, Hokkaido 078-8510 Japan

**Keywords:** Microphysiological system, Mesenchymal stem cell, Therapeutic angiogenesis, Mechanosensing

## Abstract

**Background:**

Sprouting angiogenesis is an important mechanism for morphogenetic phenomena, including organ development, wound healing, and tissue regeneration. In regenerative medicine, therapeutic angiogenesis is a clinical solution for recovery from ischemic diseases. Mesenchymal stem cells (MSCs) have been clinically used given their pro-angiogenic effects. MSCs are reported to promote angiogenesis by differentiating into pericytes or other vascular cells or through cell–cell communication using multiple protein–protein interactions. However, how MSCs physically contact and move around ECs to keep the sprouting angiogenesis active remains unknown.

**Methods:**

We proposed a novel framework of EC–MSC crosstalk analysis using human umbilical vein endothelial cells (HUVECs) and MSCs obtained from mice subcutaneous adipose tissue on a 3D in vitro model, microvessel-on-a-chip, which allows cell-to-tissue level study. The microvessels were fabricated and cultured for 10 days in a collagen matrix where MSCs were embedded.

**Results:**

Immunofluorescence imaging using a confocal laser microscope showed that MSCs smoothed the surface of the microvessel and elongated the angiogenic sprouts by binding to the microvessel’s specific microstructures. Additionally, three-dimensional modeling of HUVEC–MSC intersections revealed that MSCs were selectively located around protrusions or roots of angiogenic sprouts, whose surface curvature was excessively low or high, respectively.

**Conclusions:**

The combination of our microvessel-on-a-chip system for 3D co-culture and image-based crosstalk analysis demonstrated that MSCs are selectively localized to concave–convex surfaces on scaffold structures and that they are responsible for the activation and stabilization of capillary vessels.

**Supplementary Information:**

The online version contains supplementary material available at 10.1186/s13287-022-03223-1.

## Introduction

Sprouting angiogenesis is an important mechanism for morphogenetic phenomena, including organ development, wound healing, and tissue regeneration [1, 2]. In regenerative medicine, therapeutic angiogenesis is a clinical solution for several ischemic diseases like ischemic heart and peripheral arterial diseases [3–5]. Mesenchymal stem cells (MSCs) are multipotent stromal cells distributed around many peripheral tissues, including adipose tissue and bone marrow in vivo. They have already been clinically applied to tissue regeneration, and their therapeutic effects have been evaluated through various strategies, including transplantation of the stem cells or engineered grafts such as cell sheets or administration of the secretome constituents into humans or experimental animals [6–8]. MSCs are a promising tool for future stem cell treatment. The mechanisms by which MSCs initiate and promote angiogenesis include the differentiation of MSCs into pericytes (PCs) or other vascular cells, or cell–cell interactions (CCIs) via secretion factors and adhesion molecules. Many researchers have conducted comprehensive analyses of ECs in 2D under monoculture or co-culture with other types of cells, and the molecular basis of angiogenesis has been actively discussed [9–14]. In these methodologies, the tissue structures are always disrupted via tissue homogenization or sectioning, and spatiotemporal information is inevitably missing. Accordingly, the spatiotemporal behavior of MSCs during sprouting angiogenesis remains unclear. The mechanisms of how MSCs begin to migrate and adhere to the blood vessels and keep the angiogenesis active for the maturation of the capillary blood vessels, are unclear.

While various MSCs have been widely investigated, the presence of capillary-resident multipotent stem cells has been recently reported. The capillary-resident stem cells (CapSCs) were identified as a multipotent fraction of PCs, which expresses a gene named Ephrin type-A receptor 7 (*EphA7*), along the capillary vessels in peripheral tissues, including subcutaneous adipose and skeletal muscular tissues [15, 16]. PCs are important for vascular maturation and homeostasis as investigated well at the molecular level using juxtacrine or paracrine signaling axes, including vascular endothelial growth factor (VEGF), angiopoietin-1 (Ang-1) or angiopoietin-2 (Ang-2), transforming growth factor beta (TGF-β), and platelet-derived growth factor-BB (PDGF-BB) [17–20]. Previous studies have shown that CapSCs have stronger pro-angiogenic effects during blood flow recovery in vivo than adipose tissue-derived MSCs and that they differentiate into vascular ECs and PCs and form capillary-like structures by themselves in vitro [15, 16]. However, as with other MSCs, the mechanism by which CapSCs initiate and promote angiogenesis as well as the mechanism of interaction with vascular ECs at the cellular level, remains unclear, as described above. Furthermore, it is reasonable to assume that physical factors at the cellular scale should be involved in the behavior of adherent cells around the blood vessels.

Because cells are composed of organelles like cell nucleus, cytoskeleton, and cell membrane, they have physical properties, such as adhesion and elasticity, and behave based on the physical free energy of the entire cell while sensing the surface roughness, stiffness, and adhesiveness of neighboring cells, or scaffold structure [21, 22]. For example, PCs cover blood vessels while sensing their fine morphology, i.e., surface curvature, such as branching and tortuosity, to control the vascular functions [19, 23]. MSCs change their gene regulation related to angiogenesis, such as VEGF and integrin β1, depending on the scaffold curvature [24]. Therefore, different phenomena may occur physically and biochemically between the covered and uncovered sites on the capillary blood vessel.

Given that MSCs activate therapeutic angiogenesis, it is inevitable to consider CCIs between ECs and MSCs. Among several technologies to investigate CCIs [25], single-cell techniques have been used to analyze such CCIs at the molecular or cellular level. Its comprehensiveness makes it a powerful tool to obtain additional molecular insights [10, 13]. However, given that typical protocols require enzymatic digestion of the tissue into single cells with collagenase, the morphological information received is indirect or lost at the cellular level. Although spatial transcriptomics could provide a solution to such challenges, this technology cannot be applicable to track morphological changes continuously [26–28]. Thus, it would be crucial to develop methodologies to analyze CCIs using complementary and compatible morphological information with the aforementioned single-cell techniques.

The microphysiological system (MPS) is an effective tool for mimicking the in vivo microenvironment, including CCIs. By applying 3D imaging systems in vitro, MPS allows spatiotemporal quantitative evaluation of multicellular phenomena associated with protein–protein interactions (PPIs) and morphological changes such as angiogenesis at the cellular level. Through the MPS, various factors, including location, density, and intensity of cells, ECM, or other components, can be controlled and visualized in space and time [29–32]. For example, we have previously shown angiogenic effects of VEGF stimulation [33, 34] or co-culture with PCs [20] or senescent fibroblasts [35] on our microvessel-on-a-chip system.

In this study, we established a 3D co-culture system using a microvessel-on-a-chip to evaluate the contribution of the MSCs derived from mouse adipose tissues to sprouting angiogenesis. We analyzed the 3D vascular morphologies using our microvessel-on-a-chip model and confocal laser microscopy. Three-dimensionally reconstructed structures were analyzed to calculate several morphological indices, including object volume, surface area, and curvature. Under co-culture conditions with the MSCs, the microvessels increased the parent vessel diameter, the sprout length, and the sprout surface smoothness, allowing the evaluation of the maturation effects of the MSCs on the microvessels. Moreover, (1) MSCs migrated toward and adhered to the microvessel immediately upon co-culture and stayed around during co-culture; (2) MSCs were more likely to be localized around protrusions, along the sprouts, or at the root of sprouts than flat surfaces; and (3) microvessels became smoothed, i.e., morphologically mature. Altogether, a part of the mechanism in therapeutic angiogenesis has been revealed at the cellular level, suggesting that the MSCs activate sprouting angiogenesis and maintain its activity using their cellular behavior: migration and binding to the specific surfaces on capillary blood vessels. Furthermore, a PPI analysis using a public RNA sequencing (RNA-seq) dataset showed that the temporal changes in cellular function might be associated with cell-to-cell communication between ECs and MSCs. We first demonstrated a combination of 3D imaging and comprehensive gene expression analyses to recapitulate the EC–MSC crosstalk during sprouting angiogenesis.

## Materials and methods

### Isolation of MSCs from mice

Transgenic mice (C57BL/6J background) which ubiquitously express the green fluorescent protein (GFP) gene were kindly donated from Dr. Masaru Okabe (Osaka University, Japan) and used as described previously [16]. These transgenic GFP-expressing mice were housed under specific pathogen-free (SPF) conditions and kept at 20–26 °C under a 12 h:12 h light–dark cycle. In this study, after anesthesia with 2% isoflurane inhalation, five male mice (12–16 weeks of age) were used for the preparation of MSCs. All animal procedures were approved by the Animal Care and Use Committee of Asahikawa Medical University.

Two types of MSCs were used in this study: CapSCs and adipose-derived stromal cells (ASCs). CapSCs and ASCs were isolated from the subcutaneous adipose tissues of GFP-expressing mice as previously described [16]. Briefly, CapSCs were isolated from *EphA7*-expressed pericyte fractions with multipotency, while ASCs were crudely isolated.

### Cell culture

#### HUVECs

Human umbilical vein endothelial cells (HUVECs, Catalog #C2519A, Lot #0000699241; Lonza, Basel, Switzerland) were cultured in an Endothelial Cell Growth Medium-2 BulletKit (EGM-2; Lonza, Basel, Switzerland). The cells between passages 4 and 7 were used in this study.

#### MSCs (CapSCs and ASCs)

CapSCs were cultured in DMEM, low glucose, GlutaMAX™ Supplement, pyruvate (Gibco, Thermo Fisher Scientific, Waltham, MA, USA), containing 4% fetal bovine serum (FBS; Biosera, Nuaille, France), Insulin-Transferrin-Selenium-Sodium Pyruvate (ITS-A) (100×) (Gibco, Thermo Fisher Scientific, Waltham, MA, USA), penicillin–streptomycin (FUJIFILM Wako, Osaka, Japan), 0.05% bovine serum albumin (BSA) (Sigma-Aldrich, St. Louis, MO, USA), 20 ng/mL Recombinant Murine EGF (PeproTech, New Jersey, USA), and 20 ng/mL Recombinant Murine FGF-basic (PeproTech, New Jersey, USA). ASCs were cultured in DMEM (low glucose) (FUJIFILM Wako, Osaka, Japan) supplemented with 10% FBS (Biosera, Nuaille, France). HUVECs, CapSCs, and ASCs were seeded on tissue culture polystyrene dishes (Corning, NY, USA) and incubated in a 5% CO_2_ atmosphere at 37 °C.

### Microvessel-on-a-chip co-cultured with MSCs

To prepare a 3D microvessel with CapSCs in its collagen gel microenvironment, the polydimethylsiloxane (PDMS)-based chips (25 mm × 25 mm × 5 mm: width × length × height) were used as previously described [35]. To prevent the detachment of the collagen gel from the PDMS surface during cell culture, the surface modification was performed as follows. PDMS chips were treated with O_2_ plasma, and 3-aminopropyl trimethoxysilane (Sigma-Aldrich, St. Louis, MO, USA) was coated with 2.5% glutaraldehyde by the vapor method under reduced pressure for 30 min. Furthermore, cold neutralized collagen solution (Cellmatrix Type I-A collagen solution, Nitta Gelatin, Osaka, Japan) was prepared as the manufactured protocol.

CapSCs or ASCs containing collagen solution (40 µL, 2.5 × 10^5^ cells/mL) were introduced into the PDMS chip, and the BSA-coated acupuncture needle (200 μm in diameter) (No. 08, J type; Seirin, Shizuoka, Japan) was inserted through the PDMS channel. First, the chips were incubated at 37 °C in 5% CO_2_ atmosphere for 60 min to allow gelation of collagen solution, then warm 1-mL MSC media was added to the chip. The acupuncture needles were gently removed the next day. Subsequently, the HUVEC suspension (6 µL, 1 × 10^7^ cells/mL) was introduced into the channels, followed by coating with fibronectin (10 µL, 1 mg/mL), and the cells were let to attach onto the collagen luminal surface at 37 °C for 10 min. Warm EGM-2 (1 mL) was then added and cultured at 37 °C in 5% CO_2_ atmosphere. The medium change with EGM-2 was performed every day.

### Fluorescent staining of cells

For the live imaging of the microvessels, HUVECs were fluorescently stained with 20 µg/mL rhodamine-conjugated Ulex Europaeus Agglutinin I (UEA I) (Vector Laboratories, Burlingame, CA, USA). For the imaging of fixed samples after 10-day culture, the cells were fixed with 4% paraformaldehyde (PFA) (FUJIFILM Wako, Osaka, Japan) in D-PBS(-) (FUJIFILM Wako, Osaka, Japan) overnight at 4 °C, and permeabilized with 0.5% Triton X-100 (Sigma-Aldrich, St. Louis, MO, USA) in PBS for 10 min at room temperature. The cells were treated with a blocking solution, 1% BSA in D-PBS(−), overnight at 4 °C. They were then incubated with a primary antibody against CD31 (1:200) (Dako, Agilent Technologies, Santa Clara, CA, USA) in the blocking solution overnight at 4 °C. After several washes with D-PBS(−), cells were incubated for 2 h at 25 °C or overnight at 4 °C with goat anti-mouse IgG (H + L) cross-adsorbed secondary antibody, Alexa Fluor 555 (1:200) (Invitrogen, Thermo Fisher Scientific, Waltham, MA, USA). Additionally, nuclei were stained with Hoechst 33342 (Invitrogen, Thermo Fisher Scientific, Waltham, MA, USA) by incubating the cells for 15 min at 25 °C.

### Time-dependent crosstalk between microvessels and MSCs (2D image analysis)

#### Microscopy

To analyze cell-to-cell interaction of microvessels and CapSCs during sprouting angiogenesis, 2D microscopic images of fluorescently labeled microvessel monoculture (*n* = 1) and co-culture (*n* = 1) samples were captured from day 1 to day 10 using Axio Observer Z1 (Carl Zeiss, Oberkochen, Germany), with a 20× objective lens (LD Plan-NeoFluar 20×/0.4 Korr Ph2 M27) and ZEN 2 blue edition software (version 2.0.0.0, Carl Zeiss).

#### Spatial correlation analysis between microvessels and CapSCs

To quantify the correlation between the two signal images from UEA I (in red) as HUVECs and GFP (in green) as CapSCs, we divided the two signal images into small-kernel windows with 50 × 50 pixels (44.5 μm × 44.5 μm) and calculated the density of red and green intensities. Then, we changed the kernel window size and confirmed that larger window sizes correspond with a higher correlation between the red and green intensities. Finally, these datasets were expressed as a density map and scatter plot showing a correlation in fluorescence intensity of HUVECs and MSCs.

#### Location and orientation analysis between microvessels and MSCs

To compare angle distributions of “neighbor” MSCs and “distant” MSCs, we first extracted the vascular edges of the parent vessel from the red images. After background subtraction, Gaussian filtering, and band-pass filtering along the horizontal axis using fast Fourier transform, modified Otsu’s algorithm was used to binarize the images [36]. Then, for each side of the upper and lower vascular edges, the most distant binarized pixels along and from the center of the parent vessel were considered to represent the parent vessel’s edges. Next, we processed the green images to characterize the spatial distribution of MSCs. Background signals were subtracted using the ImageJ plugin. Similar to the red images, the green signals were binarized using the Otsu filter [36].

Particle analysis was applied to the binarized images using ImageJ, to estimate the orientation of MSCs. Given that the features were computed by fitting ellipses to binarized objects, the tilt angles were regarded as MSC orientations in the range of 0°–90°. Since the parent vessel surfaces were deformed and the MSCs migrated on the vascular surface during the 10-day culture, some green signals appeared inside the lumen, and a few objects were extracted even in the intraluminal center region in 2D images. In this study, we only analyzed the green objects beside or outside the vascular edges. Furthermore, a few green objects were surrounded by the vascular edges even on day 1. Therefore, we considered that such green objects were already in touch with the lumen wall.

We adopted the ellipse locations of such objects inside the lumen on day 1 as an intraluminal margin to define the area of “neighbor” MSCs. We also set an extraluminal margin whose distances from the edge are similar to the intraluminal margin. We regarded the ellipse objects surrounded by the two margin lines on day 1 as “neighbor” MSCs and the rest of the extraluminal objects as “distant” MSCs. Even after day 2, we used the same margin distances for the corresponding chips until day 10. Tilt angle distributions of “neighbor” and “distant” MSCs were compared with directional charts.

### Distribution of MSCs and morphology of microvessels (3D image analysis)

#### Microscopy

Fluorescently labeled microvessel monoculture and co-culture samples on day 10 were used. Z-stack images were taken using a confocal laser scanning microscope (Laser Scanning Microscope 700, LSM 700; Carl Zeiss, Oberkochen, Germany) equipped with 20× and 40× objective lens (Plan-Apochromat 20×/0.8 and LD C-Apochromat 40×/1.1 W Korr M27, respectively). Red, green, and blue fluorescence were detected using lasers whose wavelengths were 555, 488, and 405 nm, respectively.

#### Image processing

The pictures were processed using a 3D median filter in Zen software and then binarized with IMARIS (version 9.0.0, BitPlane, Zurich, Switzerland) with the same parameter settings among the samples. We extracted the parent vessel structure connected with the sprouts for each sample, in order to focus on the shapes of the parent vessels and angiogenic sprouts branching from the parent vessels. Green channel stack images were binarized using IMARIS to determine the binding sites of MSCs on the microvessel surfaces. By allocating all vertices in the 3D microvessel model into the voxel coordinates, each vertex was tagged as “inside” or “outside” of MSCs, depending on whether each green intensity was positive or zero.

#### Spatial correlation analysis between microvessels and MSCs

To quantify the correlation between the two signals from UEA I (in red) and GFP (in green), a similar analytical method was used as described in method “Fluorescent staining of cells.” After the fragmentation of z-stacked images into density maps, correlations between red and green intensity are shown as scatter plots.

#### Analyses of morphological parameters (sprout range; surface area; volume; and surface curvature)

In calculating some morphological parameters, we used geometric information on the 3D model vertices: samples with monoculture (*n* = 4) and co-culture (*n* = 5). Sprout range was defined as a spatial distance between the upper and lower sprout tips along the *Y*-axis. Surface areas on a 3D model were calculated as the summation of triangle areas of the triangular meshes. The volume of a 3D model was calculated as the sum of the outer products using the vertex coordinates of the triangular meshes. The volume surrounded by the generated 3D model of the neovascularization was calculated as the volume of the neovascularization. Then, to make the surface area of the 3D model on the neovascularization surface area, the voxel region outside the parent vessel defined earlier was eroded by ten pixels, and compartmentalization was conducted, similar to the procedure described above. The 3D model region was determined to belong outside the parent vessel and considered to be the neovascularization surface, and the surface area was calculated. The 3D model’s surface area was divided by the volume, used as an index of the surface roughness. After exporting 3D models from the IMARIS software, mean surface curvature distributions were computed using MeshLab (version 2021.10) and visualized using Blender (version 2.83.19.0).

### Statistics

To quantitatively evaluate the co-localization of HUVECs and CapSCs in 2D microscopy, one representative sample from each HUVEC monoculture and HUVEC-CapSC co-culture was used to calculate Pearson’s correlation coefficient, *r,* for each image. If the *r* values were > 0.6, we considered the datasets correlated with each other. Additionally, to characterize CapSC orientations depending on time and distance from the parent vessel in 2D, we prepared one co-culture sample and conducted a two-way analysis of variance (ANOVA) followed by the Bonferroni’s post hoc tests.

To evaluate vascular morphology and CapSC-binding sites, we analyzed four samples from the HUVEC monoculture and five from HUVEC-CapSC co-culture. The same samples were analyzed to exhibit the multiple aspects of vascular morphologies and CapSC behavior depending on the purposes and types of image processing. To compare vascular morphologies (sprout ranges, surface area, and volume), we conducted Mann–Whitney U tests. *p* values from the tests were adjusted with Bonferroni’s multiple corrections. Two-sampled Kolmogorov–Smirnov tests were used to compare surface curvature distributions. All statistical differences were considered significant when *p* values were < 0.05.

### Identification of PPIs between ECs and MSCs

To understand cell-to-cell communication at the molecular level between ECs and MSCs, we analyzed a transcriptome dataset obtained from experiments wherein HUVEC was co-cultured with MSC derived from human adipose tissues, which are publicly available [9], to estimate the temporal changes in cellular functions. RNA sequencing was performed under three conditions: (1) HUVEC monoculture; (2) MSC monoculture; and (3) HUVEC–MSC co-culture for 12 or 24 h. Gene expression profiles were normalized (mean = 0, standard deviation = 1) and transformed into log-twofold changes against control. Up-regulated or down-regulated genes with log-twofold change > 1 or < − 1 were identified as differentially expressed genes (DEGs), respectively. The DEGs were subjected to a hierarchical clustering for ECs and MSCs with cosine distances among the vectors and Ward’s method [37] to reduce the variance within each cluster. The numbers of the clusters were decided such that the correlation coefficients within each cluster were > 0.6. To categorize the gene functions in each cluster, gene ontology (GO) terms were extracted and summarized using the DAVID knowledgebase [38, 39]. According to the temporal patterns, all DEGs were classified into five up-regulated clusters, EC-U1, EC-U2, MSC-U1, MSC-U2, and MSC-U3, or four down-regulated clusters, EC-D1, EC-D2, MSC-D1, and MSC-D2. We estimated key PPIs between ECs and MSCs using a database, STRING (https://string-db.org). Using a few indicators of confidence (database, experimental, and text-mining scores) provided from the database, molecular interactions with poor confidence were screened out from the PPIs returned. Furthermore, in this study, we did not consider DEGs that were changed in both ECs and MSCs. Finally, a few key PPIs of high confidence (all the scores > 0.9) were identified. PPIs were visualized using the free software, Cytoscape (version. 3.9.1).

## Results

### MSC behaviors dynamically regulate sprouting angiogenesis

**Fig. 1:**
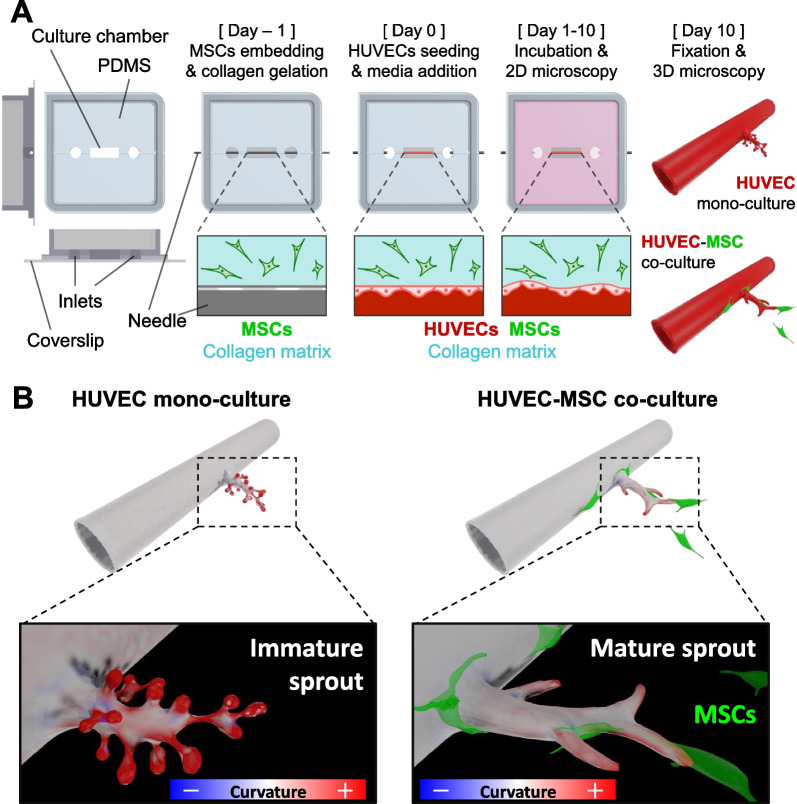
Conceptual sketch of this study to analyze angiogenic effects of MSC co-cultured on the microvessel-on-a-chip system. **A** Experimental procedure of microvessel fabrication. MSCs were embedded in the collagen gels to achieve the microvessel-on-a-chip surrounded by MSCs. Analyses of cell-to-cell crosstalk between microvessels and MSCs during sprouting angiogenesis are performed. **B** Analytical overview to evaluate the morphological maturation of angiogenic sprouts using surface curvature. Color bars represent the mean curvature calculated on the 3D model surfaces. Gradient color bars with blue, white, and red indicate convex surface, flat surface, and concave surface, respectively

To evaluate the contribution of MSCs to morphological changes in the angiogenic sprouts at the cell-to-tissue level, we fabricated microvessel-on-a-chip using HUVECs in a collagen matrix where CapSCs as MSCs were embedded (Fig. 1A). HUVEC–MSC co-culture system accelerated sprouting angiogenesis without additional VEGF and formed elongated mature sprouts. By performing the 2D or 3D imaging analytical methods in this study, we quantified the morphological maturity index of the microvessels, such as surface curvature (Fig. 1B).

**Fig. 2:**
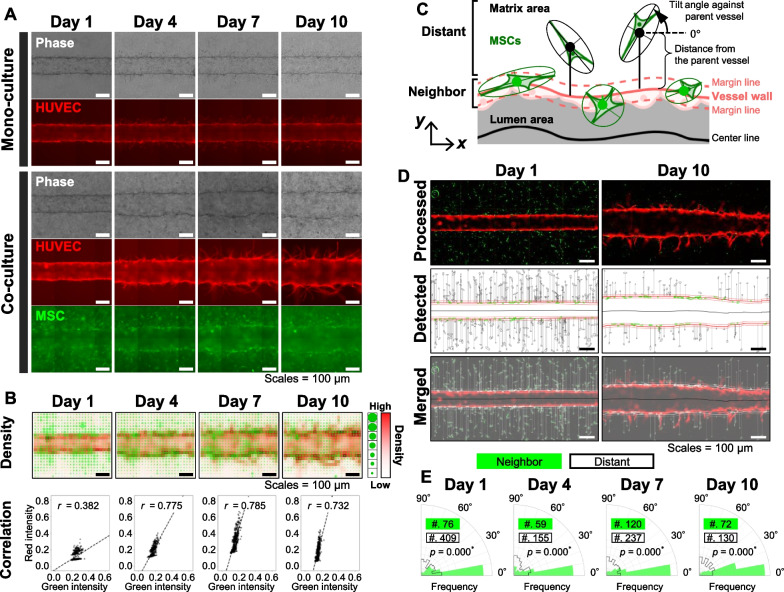
Dynamic-sprouting angiogenesis synchronized with MSC migration and adhesion. **A** Representative 2D fluorescent microscopic images in time series during mono- and co-cultures for 10 days. Monoculture did not show the clear angiogenic sprout, whereas co-culture with MSCs accelerated the formation of mature sprouts. MSCs (green) are encapsulated in the collagen gel toward microvessels (red) and attach to the vessel walls’ surface at an early stage. The elongation of sprouts was promoted after day 4. **B** Spatial correlation analysis between HUVECs and MSCs. The distribution of HUVECs and MSCs from one co-culture sample is shown as density maps and scatter plots by analyzing the co-culture images shown in (**A**). Pearson’s correlation coefficients, *r*, were calculated for each day and are shown in the plots. The correlation was constantly high (> 0.7) after day 4. **C**–**E** Orientation analysis of MSCs around the microvessels. Scale bars: 100 μm. **C** Definitions of MSC location and orientation around microvessels to analyze their behavior around microvessels. MSCs beside the vessel walls within a marginal region were considered “neighbor” cells, while MSCs outside of the margin were considered “distant” cells. **D** Image processing in 2D to quantify the location and orientation of MSCs during co-culture using one sample. After the red channel images were processed, the microvessel’s surface edges were extracted and smoothed (red or thick white lines). After the green channel images were processed, the location and orientation of MSCs were quantified. Please note that the areas in the two pictures are from the same ROI on day 1 and day 10. See “Materials and methods” section. Scale bars: 100 μm. **E** Orientation analysis of “neighbor” or “distant” MSCs by fitting ellipses to the binarized MSC objects. Histograms with thick black lines or filled with green represent “neighbor” or “distant” MSCs, respectively. The numbers in the directional charts represent the number of objects that were detected by the particle analysis. The tilt angles of “neighbor” MSCs were constantly smaller than those of “distant” MSCs through co-culture. Statistical significances between “distant” and “neighbor” MSCs were evaluated using a set of time-lapse images from one HUVEC-CapSC co-culture sample with two-way ANOVA followed by the Bonferroni’s post hoc tests using this fluorescent image set (*n* = 1). *p < 0.05; **p < 0.01

The microvessel-on-a-chip model allowed observation of sprouting angiogenesis time-dependently induced by MSC co-culture. We found that in the HUVEC monoculture condition, tiny immature sprouts were generated from day 1 to day 10 (monoculture panels in Fig. 2A), and these sprouts broke off in the middle and had rough surfaces. Alternatively, under the MSC co-culture condition, morphologically mature sprouts with thicker or longer shapes were formed within a few days (co-culture panels in Fig. 2A). We have also confirmed that the angiogenic sprouts in the co-culture system were maintained without regression of more than 2 weeks, while HUVEC monoculture shows the detachment of cells from the collagen luminal surface (data not shown). Furthermore, time-course images show that MSCs embedded in the collagen gel appeared to migrate toward and adhered to the microvessel wall up to day 4 and continued to stay close to the parent vessel and generated sprouts until day 10 (Fig. 2A, B).

To understand the time-dependent MSC distribution around the microvessel during sprouting angiogenesis, we compared the spatial distribution of red and green channel intensities by dividing the images into small rectangular regions, expressed as a density map and scatter plot showing a correlation in fluorescence intensity of HUVECs and MSCs (Fig. 2B). The scatter plot shows that the correlation between red and green intensities changed with time. The values of Pearson’s correlation coefficient from day 1 to day 10 were 0.382, 0.604, 0.728, 0.775, 0.788, 0.818, 0.785, 0.768, 0.734, and 0.732, respectively. After day 2, the correlation substantially increased (correlation coefficient ranging from 0.6 to 0.8), suggesting that a certain number of MSCs randomly embedded in the gels migrated toward and distributed along the vessel wall.

To quantify the spatial profiles of the location and orientation of MSCs, the red and green images were also analyzed in combination. Here, we set margin lines along the extracted edges of the microvessel to consider that MSCs were located near or far from the microvessel (see Materials and Methods for the meaning of the margin lines). MSCs surrounded by two margin lines were defined as “neighbor.” In contrast, those outside of the margin area as “distant” (Fig. 2C). On day 10, the parent vessel showed a rough surface and its diameter was not constant along itself. However, MSC distribution on the parent vessel wall seemed uniform. Particle analysis of the MSC distribution revealed that the number of MSCs in the matrix decreased during co-culture (Fig. 2D) and that the “neighbor” MSCs were likely to align in the direction with significantly smaller angles against the parent vessel than “distant” MSCs (Fig. 2E).

### Three-dimensional reconstruction, segmentation, and characterization of the microvessel structures and MSC localization

**Fig. 3:**
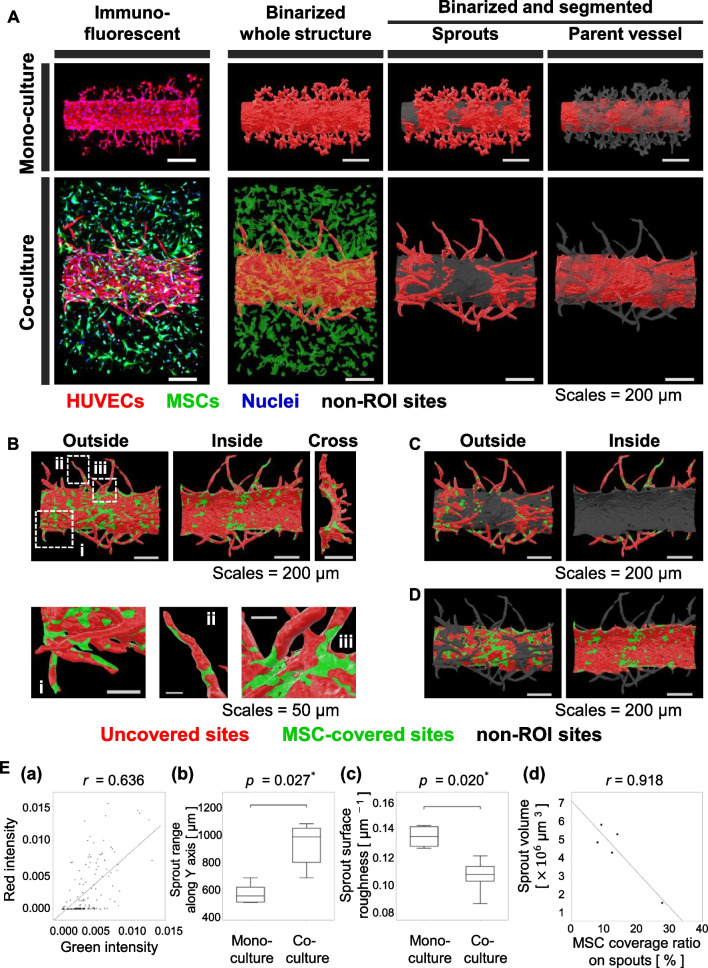
Three-dimensional reconstruction, segmentation, and characterization of microvessel morphologies when co-cultured with MSCs for 10 days. The visualization of immunostained, binarized, and segmented vascular structures from confocal laser microscopy and 3D image processing (see Additional file 1: Figs. S2 and S3; Additional file 2: Video S1; Additional file 4: Video S3). HUVECs were double-stained red with UEA I-fluorophore conjugate and immunostaining using Alexa Fluor 555-conjugated secondary antibody against CD31 marker. MSCs were labeled with GFP in green. Nuclei were stained with Hoechst 33,342 in blue. Image processing using IMARIS (Bitplane) allowed the reconstruction of 3D surface models, consisting of triangular meshes with vertices and edges. Surfaces of the 3D microvessel models were classified into “Sprouts” or “Parent vessel” (see “Materials and methods” section). Scale bars: 200 μm. **B** Segmentation of the entire surface of the 3D microvessel in (**A**) into “MSC-covered” surfaces (green) or “Uncovered” (red). Panels (i), (ii), and (iii) focus on representative MSC-binding sites as shown in white dashed boxes. Scale bars: 200 μm or 50 μm in pictures of entire or local vascular structures, respectively. **C** Segmentation of “sprout” surfaces in (**A**) into “MSC-covered” surfaces (green), “Uncovered” (red), or parent vessel (gray). Scale bars: 200 μm. **D** Segmentation of “parent vessel” surfaces in (**A**) into “MSC-covered” surfaces (green), “Uncovered” (red), or sprouts (gray). Scale bars: 200 μm. **E** Quantitative analyses of microvessel morphology. **a** Correlation analysis between red intensity (HUVECs) and green intensity (MSCs) using a single 3D image shown in (**A**). Pearson’s correlation coefficient, *r*, was calculated as high (> 0.6). **b** Comparison of sprout ranges between monoculture (*n* = 4) and co-culture (*n* = 5). Data are shown as box plots to compare the median values with the Mann–Whitney U test and are recognized as significantly different when the *p* value < 0.05. **c** Comparison of surface roughness on the sprouts between monoculture (*n* = 4) and co-culture (*n* = 5). The index “surface roughness” was defined as the unit surface area per volume. **d** Linear regression of MSC coverage ratio and sprout volume using five co-culture samples. *P* values in (**b**) and (**c**) were adjusted using Bonferroni’s multiple corrections

To understand the contribution of MSCs to the morphological maturation of sprouts, z-stacked 3D confocal images for monoculture and co-culture samples on day 10 were captured and used to be analyzed. We found that the microvessels under the co-culture condition showed expansion of the parent vessel diameter and longer elongation of the sprouts than those under the monoculture condition (Fig. 3A) (see Additional file 1: Figs. S1 and S2; Additional file 2: Video S1; Additional file 3: Video S2).

**Fig. 4:**
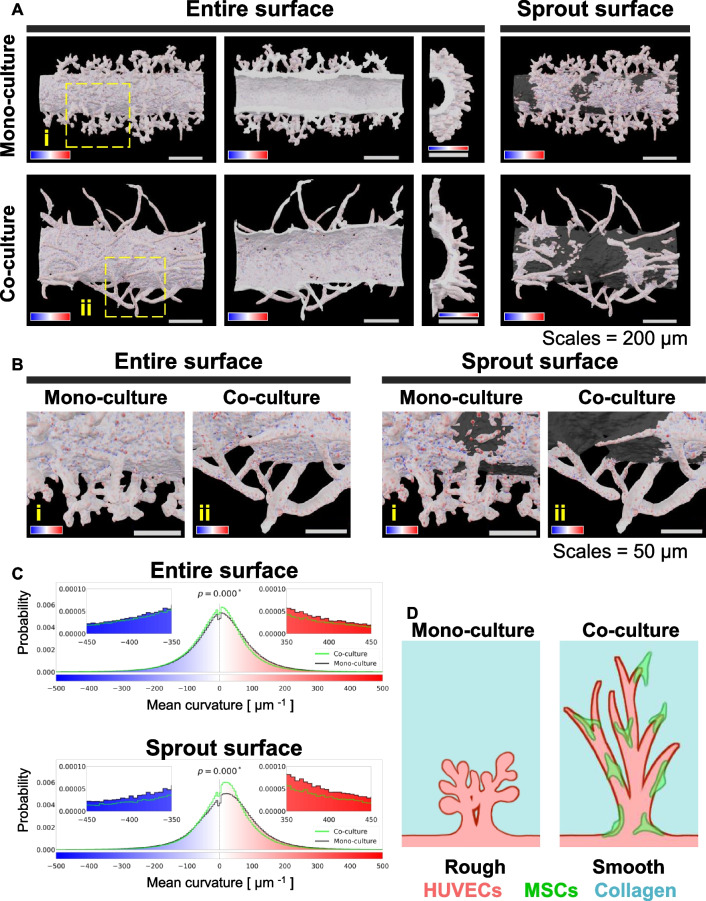
Smoothing effect of MSCs on microvessel surfaces. **A** Spatial distributions of the mean curvature on the microvessel surfaces under HUVEC monoculture or HUVEC–MSC co-culture after 10 days. Entire or segmented microvessel surfaces are colored with mean curvature. Gradient color bars with blue, white, and red indicate the mean surface curvature of negative, zero, and positive values, respectively. All ranges of the mean curvature values shown on the color bars are the same for (**A**–**C**). Scale bars: 200 μm. **B** Spatial distributions of the mean curvature on the entire microvessel surfaces. Four monoculture samples or five co-culture samples were combined to draw each histogram. The black and green lines in the graph represent monoculture and co-culture samples, respectively. Scale bars: 50 μm. **C** Histograms of the mean curvature on the segmented sprout surfaces. Similar to (**B**), datasets were combined as one, and the line colors in the graph represent culture conditions. *p* values in panel **C** were from two-sampled Kolmogorov–Smirnov tests. **D** Scheme of the maturation effect of MSCs on the microvessel sprouts

To determine spatial differences in microvascular maturation, we classified the entire microvessel surface into the “parent vessel” and “sprouts” (see Additional file 4: Video S3). Briefly, we generated a 3D microvessel surface model from a red z-stacked image of HUVECs and obtained positional information on vertices in the triangular mesh. Next, we defined the corresponding binarized region outside the microvessel using the software. By overlapping these two processed images, we were able to discriminate whether each vertex in the 3D model was included in the binarized region or not (Figs. 3, 4, and Additional file 1: Figs. S1, S3, and S4). Consequently, the sprout shapes of the co-cultured microvessels were more elongated, and the sprout surfaces were smoothed than those of monoculture (Fig. 3A).

Moreover, to characterize the MSC-dependent morphological maturation, we segmented the entire surface into the “MSC-covered” sites and the “uncovered” sites using the 3D-reconstructed structures (Fig. 3B). MSCs were likely to bind to specific sites like protrusion tips or sprout roots (Fig. 3Bi–iii) and appeared to distribute widely on the outside and partly inside of the parent vessel and sprouts (Fig. 3C). Further, 3D visualization of MSC localization demonstrated that MSCs might be merged with the parent vessel wall (Fig. 3D; see Additional file 1: Fig. S2 and Additional file 3: Video S2), implying the transdifferentiation of MSCs into ECs after adhering to the capillary surface. However, further investigation should be required.

Similar to the correlation analysis in 2D (Fig. 2B), spatial distributions of red and green intensities in 3D were correlated with each other, suggesting that GFP-labeled MSCs were localized around the microvessel (Fig. 3E-a). Utilizing IMARIS functions to generate 3D surface models consisting of a triangular mesh, we evaluated a few geometric indexes: reaching distance along the *Y*-axis, surface area, surface volume, and surface roughness. Microvessels of the angiogenic sprouts under co-culture conditions were more elongated and smoothed than those under monoculture conditions (Fig. 3E-b and c). With the surface area and volume calculation of the sprouts, we were able to estimate the coverage ratios of MSCs to the microvessels and the coverage dependency of the sprouts for morphological maturation, suggesting that MSCs tightened angiogenic sprouts (Fig. 3E-d).

### Smoothing and elongation effects of MSC co-culture on angiogenic sprouts

Previous studies have shown the behavior of adherent cells, including MSCs, on concave–convex surfaces depending on the scaffold surface structure [21–24]. The microvessels co-cultured with MSCs exhibited more mature morphologies (smoothed and elongated) than those under monoculture. Thus, we considered the surface curvature of the microvascular structure as a maturity index and quantitatively compared the surface curvature between the mono- and co-cultured microvessels. Using the open-source software, MeshLab, we estimated spatial curvature distributions on the 3D discrete surface models generated using IMARIS (Fig. 4A). We found that the microvessel surfaces had an extensive range of curvature values and that these values change depending on specific sites, such as protrusions and roots (Fig. 4B). The microvessels under monoculture conditions appeared to form sprouts with a rougher surface than those under co-culture conditions (Fig. 4B). The segmentation of the “parent vessel” and “sprouts” for the monoculture samples was not as good as the segmentation for the co-cultured samples, because the monocultured parent vessel had rougher surfaces than the co-cultured parent vessel. This may be attributable to a maturity regulation defect. We obtained spatial curvature profiles and statistically compared histograms of surface curvature between the two conditions (Fig. 4C).

Consequently, curvature histograms from the entire surface under the two conditions changed statistically. The median (2.07 μm^−1^) of the mean curvature of co-cultured samples was more concentrated to 0-μm^−1^ (flat or saddle surface) than that (7.65 μm^−1^) of the monocultured samples, suggesting that MSCs have a smoothing effect on the microvessels (Fig. 4B). The smoothing effect was more crucial for segmented sprout surfaces (Fig. 4C). These results show that MSCs tightened up the sprout surfaces during co-culture (Fig. 4D). Another co-culture experiment of HUVECs and ASCs was conducted to clarify the angiogenic effects of MSCs (Additional file 1: Fig. S5), which were crudely isolated from mouse adipose tissues. Unlike CapSCs, ASCs smoothed the surface of the parent vessel but could not promote capillary sprouting, suggesting that CapSCs possess the ability of vascular smoothing and elongation.

### Curvature-oriented behavior of MSCs on the microvessel surfaces

**Fig. 5:**
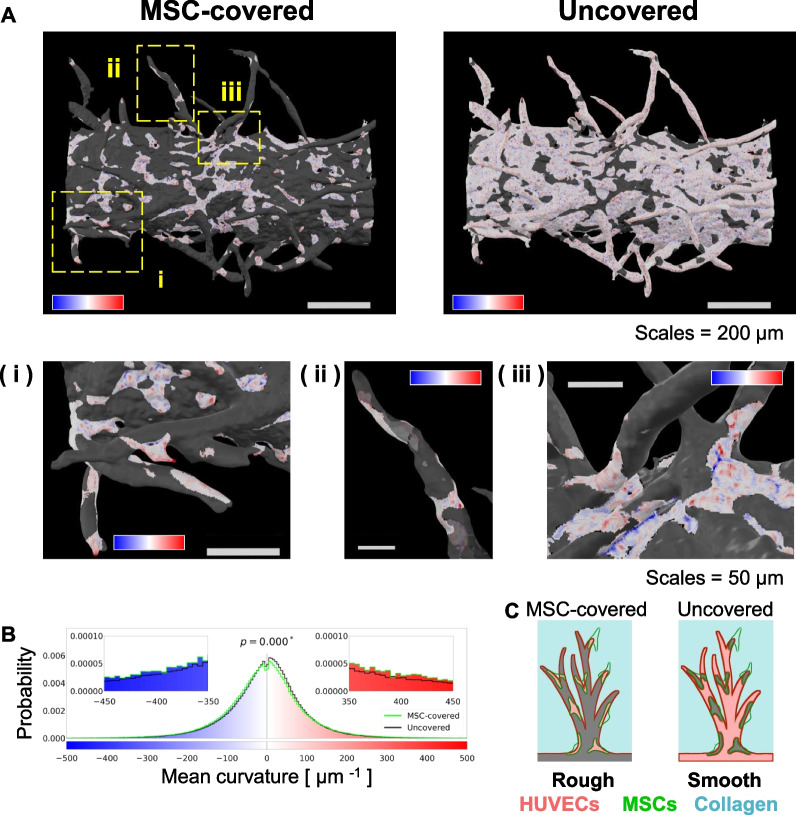
Selective MSC localization on the microvessel surface under co-culture conditions. **A** Visualization of MSC-binding sites and mean curvature on an entire microvessel surface under co-culture conditions. Gradient color bars with blue, white, and red show the mean surface curvature of negative, zero, and positive values, respectively. All ranges of the mean curvature values shown on the color bars are the same for (**A**–**C**). Scale bars: 200 μm or 50 μm in pictures of entire or local vascular structures, respectively. **B** Histograms of the mean curvature calculated on co-culture samples. Curvature values from five co-culture samples were combined as one to draw each histogram. The green and black lines in the graph represent MSC-covered and uncovered surfaces, respectively. The *p* value in the graph was from a two-sample Kolmogorov–Smirnov test. **C** Scheme of the maturation effect of MSCs on the microvessel sprouts

To gain insights into the mechanism by which the angiogenic sprouts under co-culture conditions were elongated and smoothed, we examined MSC localization on the surface of the microvessel (Fig. 5). In general, MSCs promote and maintain active angiogenesis using soluble factors (paracrine signaling), physical contact (juxtacrine signaling, adhesion, and contraction), and differentiation by themselves into vascular endothelium. Additionally, PCs are likely to bind around branching points on capillary beds in vivo*,* such as the retina, to control the vascular functions [17–19]; thus, we focused on physical cell–cell contact between HUVECs and MSCs. It is reasonable to assume that contact interactions between HUVECs and MSCs contribute directly to the maturation of the microvessel morphologies. To classify the entire microvessel surface into “MSC-covered” sites or “uncovered” sites, we developed a 3D image-based segmentation method using z-stacked images with multiple color channels. Similar to the segmentation of the parent vessel and sprouts, we generated a 3D surface model of the microvessel and obtained positional information of the vertices in the triangular mesh. Next, we binarized the corresponding green image of MSCs. By overlapping these two processed images, we could discriminate whether each vertex in the 3D model was included in the binarized region or not (Figs. 3, 5; Additional file 1: Fig. S4 and Additional file 4; Video S3). Similar to comparing the parent vessel and sprouts, we estimated and visualized spatial distributions of mean curvature on the microvessels co-cultured with MSCs (Fig. 5A). MSCs were localized to specific sites with extremely low or high surface curvature (Fig. 5B), such as the uneven surface of the parent vessel and root, middle, and tip of the new vessel (Fig. 5Ai–iii). This result indicates that MSCs promote and stabilize angiogenesis with physical contacts, as observed similarly to the behavior of PCs in vascular development [40] (Fig. 5C).

### Identifying key PPIs between ECs and MSCs.

We conducted a transcriptomic analysis to address molecular components undergoing EC–MSC crosstalk (Additional file 1: Fig. S6). In this study, we employed a public dataset of RNA-seq from co-culture experiments of ECs and MSCs, both from human tissues [9]. The dataset consisted of 2D monoculture of HUVECs or MSCs and co-culture of HUVECs and MSCs for 12 and 24 h. Gene expression profiles with log-twofold change > 1 or < − 1 were identified as DEGs. Depending on the temporal patterns (up-regulation, down-regulation, or the other), the DEGs in ECs or MSCs were classified into four or five clusters, respectively. Then, we investigated enriched GO terms for each cluster. Interestingly, the fast up-regulated clusters EC-U1 and MSC-U3 included many GO terms related to phenotypic changes, including cell migration, adhesion, and organization of the extracellular matrix (ECM), while the slow up-regulated clusters EC-U2 and MSC-U1 included many GO terms related to cell cycle and proliferation. Altogether, our microvessel-on-a-chip experiments suggest the order of cellular behavior, including migration, adhesion, ECM organization, and proliferation, is important for capillary sprouting and maturation.

Since we found no significant GO terms in clusters EC-D1, EC-D2, MSC-U2, MSC-D1, and MSC-D2, we further analyzed the molecular components of the up-regulated clusters EC-U1, EC-U2, MSC-U1, and MSC-U3. First, we searched a PPI database, STRING, to estimate the key PPIs for intercellular communication between ECs and MSCs. Then, we entered the ENSEMBL gene IDs of the up-regulated clusters into the database and obtained a list of PPIs. Because the list included low-confidence PPIs, we filtered out those of low confidence and extracted those of high confidence (see “Materials and methods” section). After the removal of PPIs that can occur inside the cell or among the same group (ECs or MSCs) from the list, three PPIs between ECs and MSCs were finally identified: bone morphology protein 2/4 (BMP2/4) to bone morphology protein receptor 1A (BMPR1A); interleukin 1 beta (IL1B) to interleukin 1 receptor type 1 (IL1R1). BMP signaling promotes angiogenesis [40–43]. IL1B signaling has been well investigated in tumor angiogenesis [44–47]﻿. As these key PPIs included DEGs grouped in fast up-regulated clusters, EC-U1 and MSC-U3, they may trigger or progress capillary growth. Along with these molecular mechanisms, MSC binding to the microvessel might have inhibited random sprouting.

## Discussion

We have established a microvessel-on-a-chip system for the 3D co-culture of HUVECs and MSCs and an image-based analytical procedure to analyze the CCIs using confocal laser microscopy. The CapSCs used as MSCs in this study showed remarkable elongated and mature sprouts; the use of CapSCs provided insight into how cells located outside blood vessels induce neovascularization and contribute to the construction of mature neovessels.

Most of the MSCs in previous studies are considered heterogeneous, making it difficult to investigate the therapeutic effects of MSCs with cell-to-cell interaction. To overcome this issue, we previously identified EphA7^+^ pericytes (PCs) called CapSCs isolated from mouse subcutaneous adipose tissue, demonstrating that (1) CapSCs differentiated into endothelial cells or PCs to form capillary-like structures by themselves, and (2) transplantation of CapSCs into ischemic tissues significantly improved blood flow recovery in hind limb ischemia mouse model compared to ASCs [16]. As shown in this study, CapSCs, derived from mouse subcutaneous adipose tissues, co-cultured with microvessels promoted sprouting angiogenesis, forming elongated and mature sprouts. However, ASCs derived from the same mouse adipose tissues did not show remarkable neovessel formation (Additional file 1: Fig. S5).

It is considered that the gradient of angiogenic signals (e.g., VEGF) initiated the angiogenic sprouts. In our previous studies, in order to induce angiogenesis in this microvessel model, it was necessary to add additional VEGF (10–50 ng/mL) to EGM-2 in both monoculture and co-culture with PCs [20, 48]. However, co-culture with CapSCs accelerated angiogenic sprouts and expansion of parent vessels without additional VEGF (Fig. 2A, day 4). We assume that the angiogenic action in this co-culture study was induced by secreted factors, such as VEGF and FGF, by CapSCs embedded in the collagen gel, creating a gradient of angiogenic signals around the microvessels. The results of our previous study confirmed that CapSCs have a remarkable secretion of growth factors, such as VEGF and FGF, compared to pericytes isolated from the same adipose tissues [16].

As mentioned in 3.1, the angiogenic sprouts observed in the co-culture with CapSCs remained stable without regression for more than 2 weeks, while the HUVEC monoculture showed a decrease in the cells within a 1-week culture (data not shown). It was obvious that neovessels formed by co-culture with CapSCs were elongated, smooth, and mature. Our previous studies confirmed that co-culture with PC results in the formation of neovessels that do not regress for 8 days [20]. This is because PCs adhere to ECs and stabilize the vessels. It is considered that the same behavior occurs in co-culture with CapSCs. Altogether, it is evident that CapSCs promote sprouting angiogenesis and stabilize the formed neovessels through their excellent angiogenic factor secretion and differentiation ability.

Two-dimensional image analyses of morphological changes in the microvessel and MSC behavior showed that MSCs migrated within a few days and continuously stayed close to the microvessels after co-culture started. However, what triggers and maintains the movements of MSCs is unclear. Further experiments are required to verify whether MSCs migrated to the microvessel chemotactically or randomly. Comparing gene or protein expression profiles between mono- and co-culture might provide molecular insight into this phenomenon. Moreover, we found that MSCs were selectively located around fine microstructures with excessively low or high surface curvature on day 10. However, the temporal behavior of MSCs during co-culture remains obscure. Tracking analysis of MSCs with confocal live imaging would help reveal whether MSCs stay at the tips or roots of the sprouts or move randomly.

This study conducted 3D confocal imaging, reconstruction, and segmentation of the microvessel and MSC localization to characterize the microvessel maturity and MSC behavior, which was impossible in 2D image analysis. The 3D image analysis first showed that the sprout surfaces became smoothed depending on the existence of MSCs, but they covered not all surfaces. Also, 2D image analyses demonstrated that the initiation of sprouting angiogenesis was synchronized with the approach of MSCs to the microvessel and suggested that MSCs were likely to stay on the microvessel surface throughout co-culture. These results indicate that maturation may not only occur while MSCs are attached but may also be kept active continuously by activating cellular junctions among HUVECs after MSCs leave. Many studies have demonstrated that mural cells control vascular permeability and maturation by binding to the vascular surface [49]. MSCs might maintain vascular maturity by moving along endothelial junctions, because PCs in zebrafish seem to show such behavior during vascular development [40]. MPS-assisted assessment of vascular permeability on our microvessel-on-a-chip system should contribute to our understanding of such phenomena. With co-culture experiments of ASCs (Additional file 1: Fig. S5) and PPI analysis (Additional file 1: Fig. S6), it may be crucial for effective and efficient angiogenesis to balance the endothelial functions, including intercellular junctions, proliferation, migration, and ECM reorganization. These questions may be answered by further co-culture experiments with MSCs embedded away from the microvessel. As a mechanism of distant cell–cell communication, some researchers have revealed that cell–ECM–cell interaction can occur in 3D culture conditions [50–52]. Although, we identified three key PPIs that might trigger distant EC–MSC crosstalk, some GO terms related to ECM organization were included in the DEG clusters (Additional file 1: Fig. S6). The combinatorial approach of traction force microscopy and stress–strain analysis would contribute to our understanding of the EC–ECM–MSC mechanical interaction.

We estimated the surface curvature to quantitatively characterize both the microvessel maturity and site-specific MSC localization. A comparison of curvature distributions between monoculture and co-culture demonstrated that MSCs had smoothing effects. The comparison between MSC-covered and uncovered sites was useful to understand where MSCs were selectively localized. These findings suggest that MSCs have mechanosensing properties for the scaffold structure, as suggested by previous studies [53, 54]. By culturing MSCs on scaffolds coated with basement membrane components, such as collagen Type-IV and laminin, it might be possible to analyze MSC motility without HUVECs. It has been reported that human MSCs show substrate curvature-dependent changes in VEGF secretion [24]. Although PC-induced vascular maturation is known, it is unclear whether it promotes angiogenesis. MSCs used in this study were isolated from the capillary-resident pericyte fraction in mouse peripheral subcutaneous adipose tissue [15, 16], and are considered to have a pro-angiogenic function in addition to the vascular maturation effect, specific for pericytes. Additionally, it has been suggested that not only one of the endothelial functions (ECM remodeling, motility, proliferation, adhesion, etc.) is essential for successful angiogenesis, but also the balanced expression of these functions is important [55]. MSCs endowed with pericyte-like maturation functions, and pro-angiogenic properties might be promising as a balancing agent of stem cell therapy.

Conventional studies of vascular morphology with 3D image analysis have shown geometric indexes, such as branching points and tortuosity, by extracting the skeletonized or centerline of vascular structures for simple detection, segmentation, or quantification [20]. This study adopted surface curvature as an index of vessel maturity by focusing on the surface shape without abstracting the morphology of 3D objects. Additionally, by interpreting the surface as a boundary between objects, this study adopted it as an index for MSC localization analysis or HUVEC–MSC interaction analysis. The microstructure segmentation method developed in this study uses raw data from 3D models generated using the image processing software, IMARIS, which might have noisy surface features (calculated curvature from) due to artifacts of *Z* slice resolution. For a more detailed geometric analysis, it is necessary to confirm the algorithms of 3D model generation and preprocessing [56]. This study also quantified the curvature of MSC-binding sites by extracting MSC-localized sites. However, we have not yet tested whether the spatial resolution of the triangular mesh on the 3D models is optimal. It may be necessary to remesh the model at the preprocessing step, considering the size of the adherent cells and the scale of irregularity of the adherent surface. With regard to the roles of microstructures involved in CCIs, micro-protrusions are used for CCIs through physical contact. For example, peg-socket structures on the interface of ECs and PCs are reported to maintain intercellular adhesion between them [57]. Dendritic spines allow neural communication at the interface of neurons [58]. Although it is possible to estimate the morphological metrics of such microstructures using 3D rendering techniques, there are few methodologies or strategies for extracting the biological meanings of the structural information.

The morphological analyses in this study provided a phenotypic understanding of the EC–MSC interaction without describing the molecular interactions. Comprehensive analysis, such as single-cell RNA sequencing (scRNA-seq) techniques, would aid the elucidation of the molecular scenarios of MSC-assisted angiogenesis. Spatial transcriptomics based on scRNA-seq is valuable in cell–cell crosstalk analysis at the tissue level [22–24]. However, this technique takes only a tissue section for measurement at a single time point, and the findings are limited. Moreover, we used mouse-derived CapSCs in this study. Since the public RNA-seq dataset used in this study was obtained from human cells, we need to isolate CapSCs from human tissues and confirm gene expression profiles using HUVECs and CapSCs in further study. Combined with such a molecular-level analysis, our image-based approach without structural destruction would contribute to a reliable understanding of morphogenetic phenomena via intercellular communication at the tissue and cell levels.

Additionally, to evaluate the ability of MSCs to recover blood flow in vivo, it is necessary to investigate effects, such as permeability and angiogenesis, on other vascular phenotypes. Although results of static co-culture of HUVECs and MSCs were shown in this study, perfusion co-culture experiments might help us understand cell-level mechanisms of blood flow recovery [16].

MPS can be used as a pharmacological assay and as a personalized prevalidation tool for various cell-based treatments, such as stem cell therapy [59–61]. As mentioned in the introduction, in the future, MPS may serve as a tool to support the decision-making of physicians or patients by presenting the success rate of stem cell transplantation or therapeutic angiogenesis. By harvesting ECs and MSCs from patients’ adipose tissues, performing prevalidation assays on MPS might be possible.

## Conclusions

CapSCs, capillary-resident MSCs, promoted angiogenesis by smoothing and elongating the angiogenic sprouts on a microvessel-on-a-chip model. Confocal microscopy showed the microvascular maturation and the selective localization of CapSCs. These phenomena might have been triggered by a few key PPIs involved in ECM organization, cellular movement, and growth among HUVECs and CapSCs. Our method is versatile and can be applied to various cell–cell crosstalk accompanied by morphological changes. Phenotypic insights from co-culture of different cell types, such as induced pluripotent stem cells (iPSCs), on the MPS, can be applied to analyze CCI mechanisms. MPS development using patient-derived cell types may contribute to translational research for personalized medicine.

## Supplementary Information

**Additional file 1: Fig. S1.** Image processing of 3D surface generation (binarization); **Fig. S2**. 3D-reconstructed microscopic images of a co-cultured microvessel taken at different magnifications; **Fig. S3.** Image segmentation process to extract the specific surface area of interest; **Fig. S4.** Image segmentation process of angiogenic sprouts connected to the parent vessel; **Fig. S5.** Smoothing effect of co-culture with another type of adipose-derived stem cells (ASCs); **Fig. S6.** Identification of key PPIs for EC–MSC crosstalk.

**Additional file 2: Video S1.** Orthographic visualization of a 3D confocal image of the co-culture condition.

**Additional file 3: Video S2.** Comparative visualization of a co-cultured microvessel at different magnifications.

**Additional file 4: Video S3.** Three-dimensional segmentation of MSC-covered sprout surfaces.

## Data Availability

The datasets and/or analyses of this study are available from the corresponding author upon reasonable request.

## Abbreviations

EC: Endothelial cell
MSC: Mesenchymal stem cell
PC: Pericyte
PPI: Protein–protein interaction
HUVEC: Human umbilical vein endothelial cell
CapSC: Capillary-resident stem cell
2D/3D: Two-/three-dimensional
CCI: Cell–cell interaction
EphA7: Ephrin type-A receptor 7
VEGF: Vascular endothelial growth factor
Ang-1/: Angiopoietin-1/2
TGF-β: Transforming growth factor beta
PDGF-BB: Platelet-derived growth factor-BB
MPS: Microphysiological system
scRNA-seq: Single-cell RNA sequencing
ASC: Adipose-derived stromal cell
GFP: Green fluorescent protein
PDMS: Polydimethylsiloxane
PBS: Phosphate-buffered saline
FBS: Fetal bovine serum
BSA: Bovine serum albumin
UEA I: Ulex Europaeus Agglutinin 1
LSM: Laser scanning microscope
ANOVA: Analysis of variance
DEG: Differentially expressed gene
GO: Gene ontology
DAVID: Database for annotation, visualization and integrated discovery
ECM: Extracellular matrix
BMP2/4: Bone morphology protein 2/4
BMPR1A: Bone morphology protein receptor 1A
IL1B: Interleukin 1 beta
IL1R1: Interleukin 1 receptor type 1
iPSC: Induced pluripotent stem cell
