# Supplementary material for: Image-based crosstalk analysis of cell–cell interactions during sprouting angiogenesis using blood-vessel-on-a-chip
Source: Stem Cell Res Ther. 2022 Dec 27;13:532. doi: 10.1186/s13287-022-03223-1 (PMC9795717; doi:10.1186/s13287-022-03223-1)
Supplement: Supplementary file 1 — Additional file 1: Fig. S1. Image processing of 3D surface generation (binarization); Fig. S2. 3D-reconstructed microscopic images of a co-cultured microvessel taken at different magnifications; Fig. S3. Image segmentation process to extract the specific surface area of interest; Fig. S4. Image segmentation process of angiogenic sprouts connected to the parent vessel; Fig. S5. Smoothing effect of co-culture with another type of adipose-derived stem cells (ASCs); Fig. S6. Identification of key PPIs for EC–MSC crosstalk. [file 13287_2022_3223_MOESM1_ESM.pdf]

SUPPLEMENTARY INFORMATION

Image-Based Crosstalk Analysis of Cell-Cell Interactions during Sprouting Angiogenesis using Blood-Vessel-on-a-Chip

Takanori Sano [a], Tadaaki Nakajima [a, b], Koharu Alicia Senda [c], Shizuka Nakano [a], Mizuho Yamato [a], Yukinori Ikeda [a], Hedele Zeng [a], Jun-ichi Kawabe [d], Yukiko T. Matsunaga[a,*]

[a] Institute of Industrial Science, The University of Tokyo, 4-6-1 Komaba, Meguro-ku, Tokyo 153-8505, Japan

[b] Department of Science, Yokohama City University, 22-2 Seto, Kanazawa-ku, Yokohama Kanagawa 236-0027, Japan

[c] Hiroo Gakuen Junior and Senior High School, 5-1-14 Minami Azabu, Minato-ku, Tokyo 106-0047, Japan

[d] Department of Biochemistry, Asahikawa Medical University, 2-1-1 Midorigaoka-higashi, Asahikawa, Hokkaido 078-8510, Japan

* Correspondence to:

Yukiko T. Matsunaga, Ph.D.
Institute of Industrial Science, The University of Tokyo 4-6-1 Komaba, Meguro-ku, Tokyo 153-8505, Japan
Tel.: +81-3-5452–6470; Fax: +81-3-5452-6471
E-mail: mat@iis.u-tokyo.ac.jp

Table of Contents

Supplementary Figures

Figure S1. Image processing of 3D surface generation (binarization).

Figure S2. 3D-reconstructed microscopic images of a co-cultured microvessel taken at different magnifications.

Figure S3. Image segmentation process to extract specific surface area of interest.

Figure S4. Image segmentation process of angiogenic sprouts connected to the parent vessel.

Figure S5. Smoothing effect of co-culture with another type of adipose-derived stem cells (ASCs).

Figure S6. Identification of key PPIs for EC-MSC crosstalk.

1. Supplementary Figures

Figure S1.

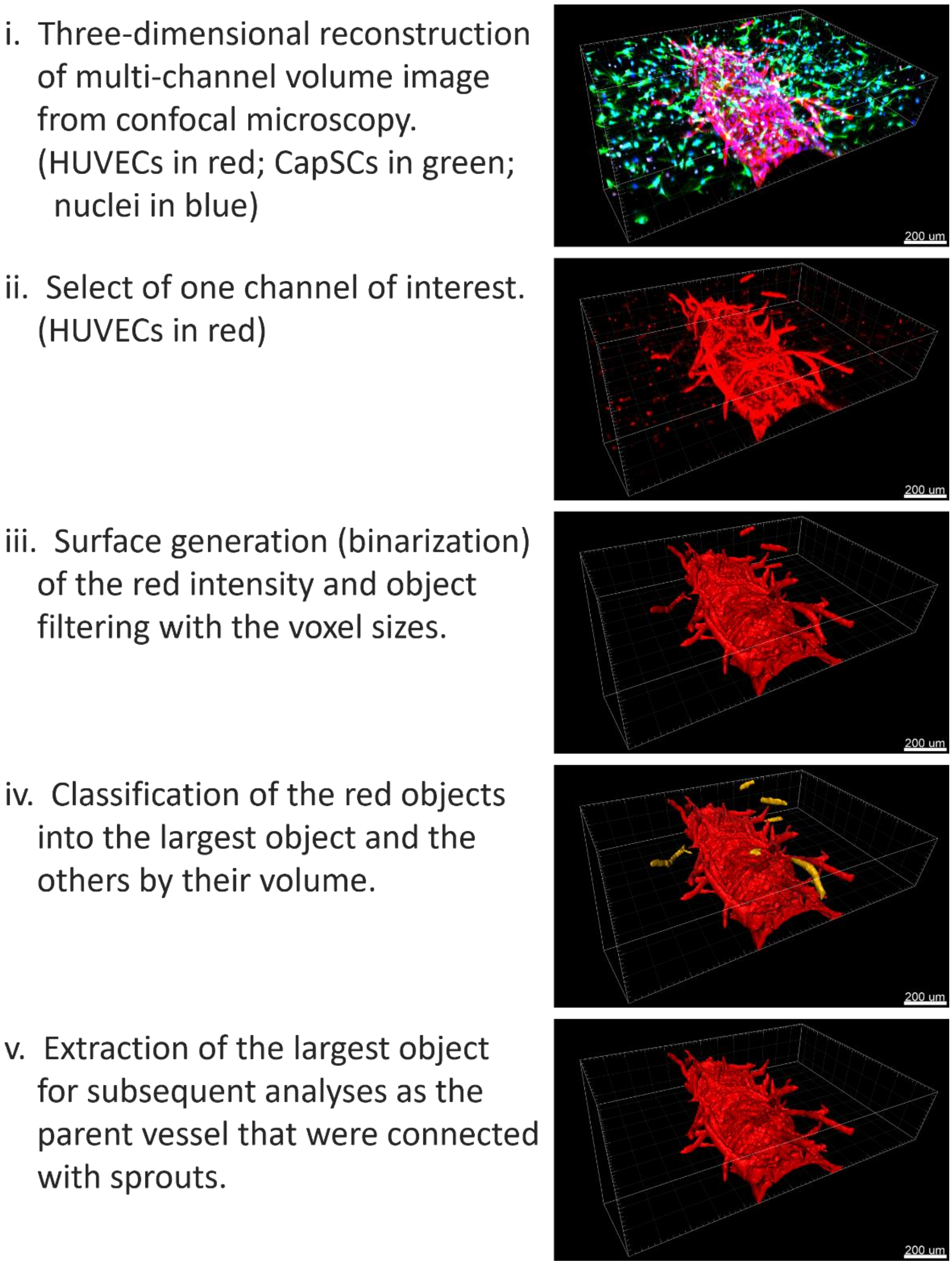

Figure S1. Image processing of 3D surface generation (binarization).

Figure S2.

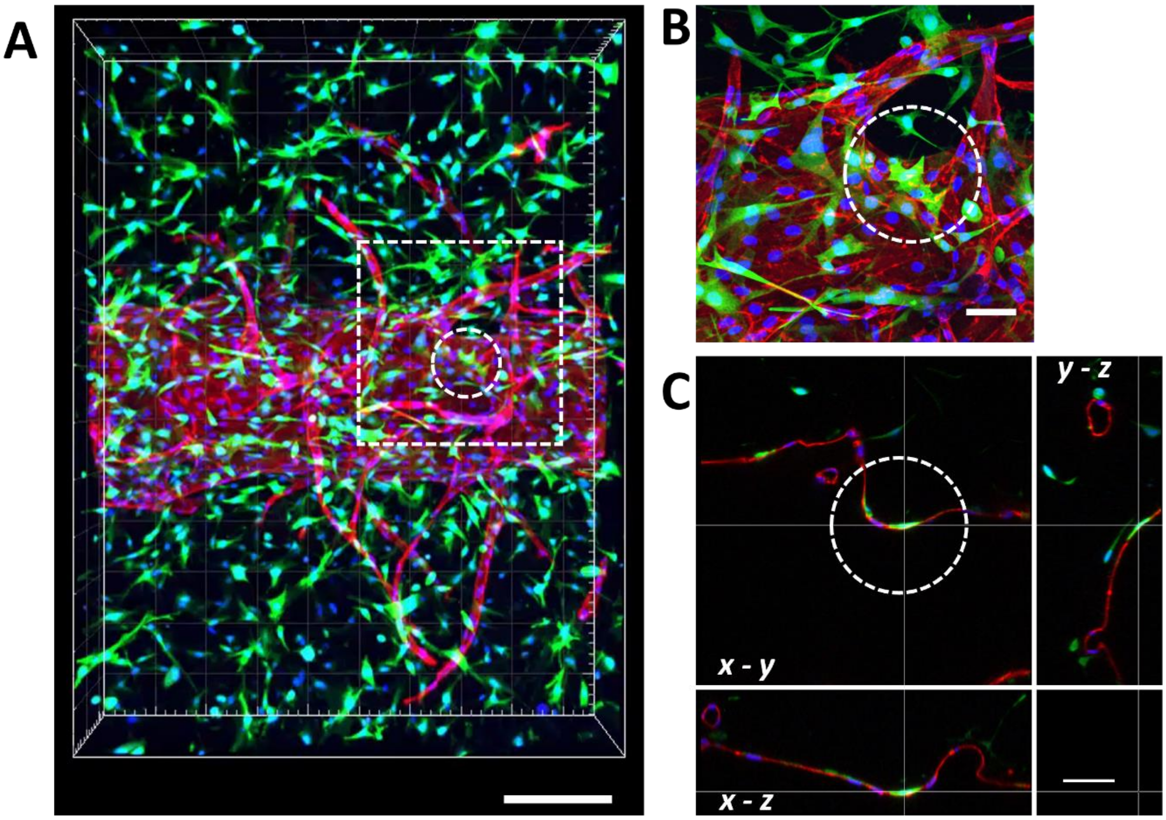

Figure S2. 3D-reconstructed microscopic images of a co-cultured microvessel taken at different magnifications. Confocal microscopy at a higher magnification illuminated the CapSCs that merged into the microvessel wall. **A**. a microscopic image at 20x magnification. Scale bar is 200 µm. **B**. a microscopic image at 40x magnification. Scale bar is 50 µm. **C**. A representative cross-sectional image focusing on the microvessel surface. Scale bar is 50 µm. HUVECs, CapSCs, and nuclei were colored in red, green, and blue, respectively. White dashed square represents the representative spot where CapSCs appeared to merge into the microvessel wall as shown on **B**. White circles represent the same position among **A**, **B**, and **C**.

Figure S3.

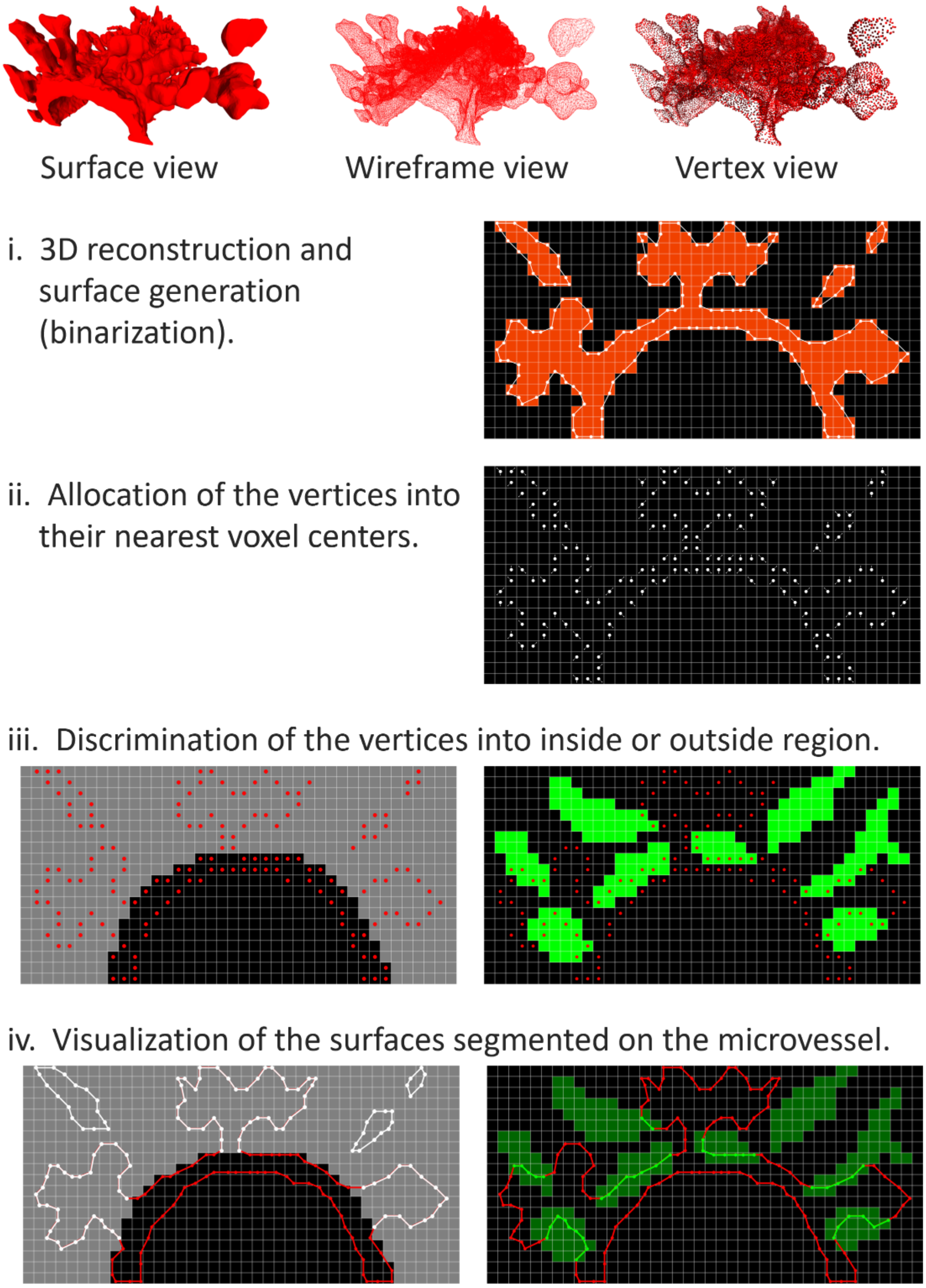

Figure S3. Image segmentation process to extract specific surface area of interest. Once a 3D surface model with triangular meshes generated (i), all the vertices were allocated to the nearest individual voxels (ii). By discriminating that each vertex is inside or outside the masked region of interest, all the vertices were considered to belong or not (iii). All the triangular meshes were colored in white or green as sprout area or MSC-covered area, respectively (iv).

Figure S4.

i. Manual definition of the external region around the parent vessel.

ii. Extraction of channel intensities in overlapped regions of the parent vessel with connected sprouts and the external region.

iii. Surface generation (binarization) of the extracted intensity and object filtering with the voxel sizes.

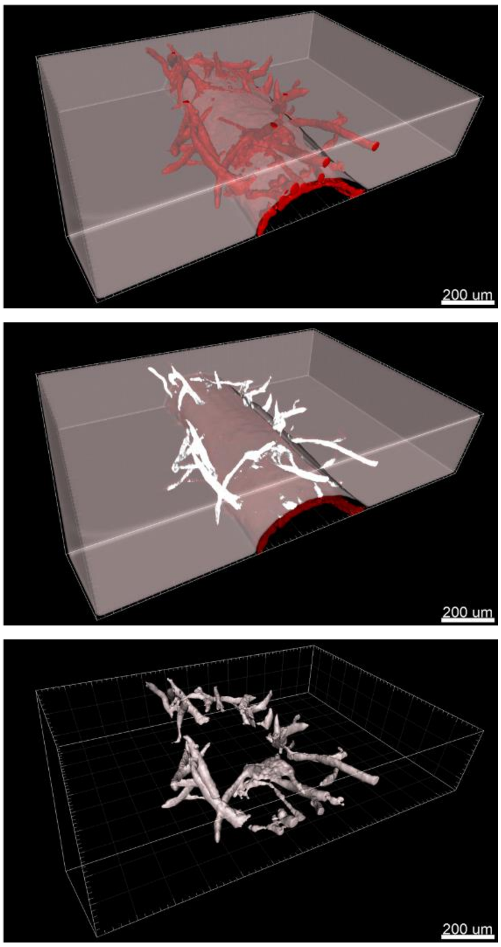

Figure S4. Image segmentation process of angiogenic sprouts connected to the parent vessel.

Figure S5.

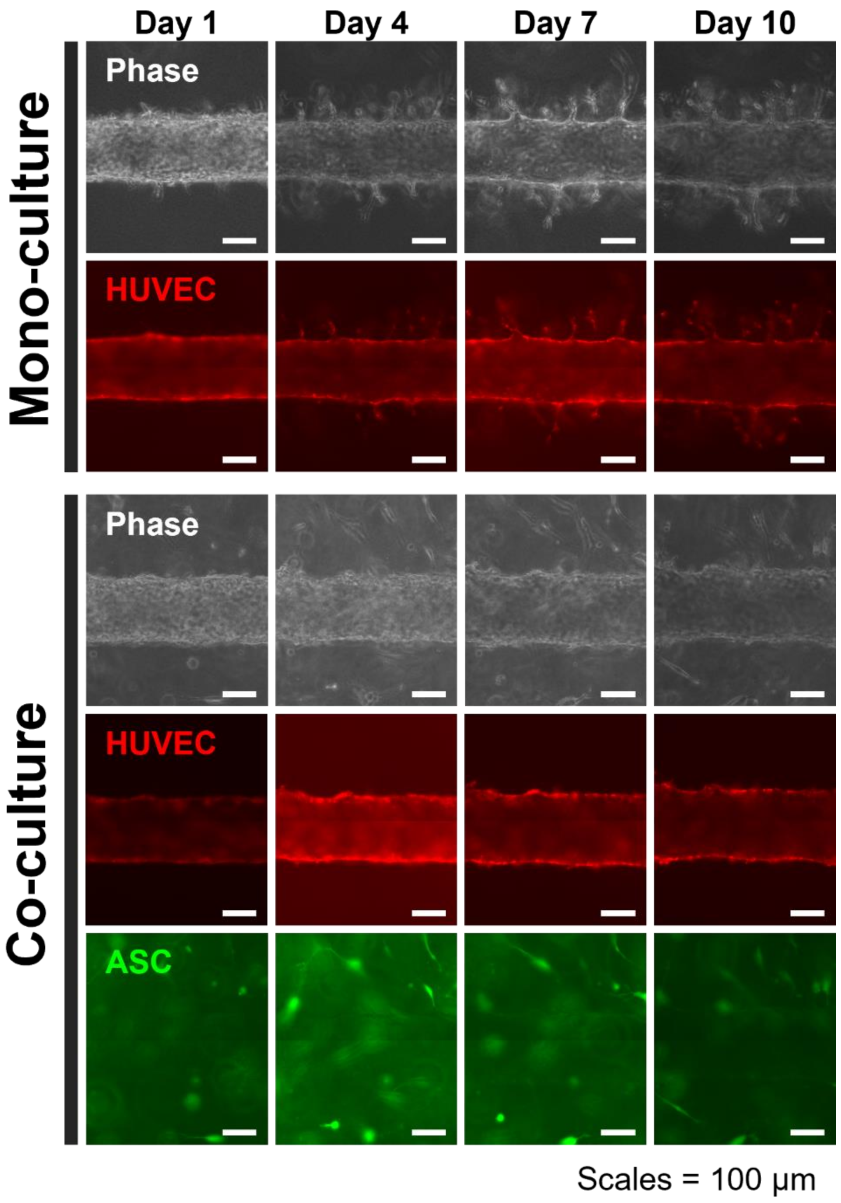

Figure S5. Smoothing effect of co-culture with another type of adipose-derived stem cells (ASCs). Microvessels were co-cultured with a subtype of crude MSCs, which were termed ASCs in this study, in the same experimental conditions as with CapSCs. In the existence of ASCs, there were no capillary sprouts generated for 10 days. HUVECs were stained with UEA I. ASCs were labeled with GFP. Scale bars: 100 μm.

Figure S6.

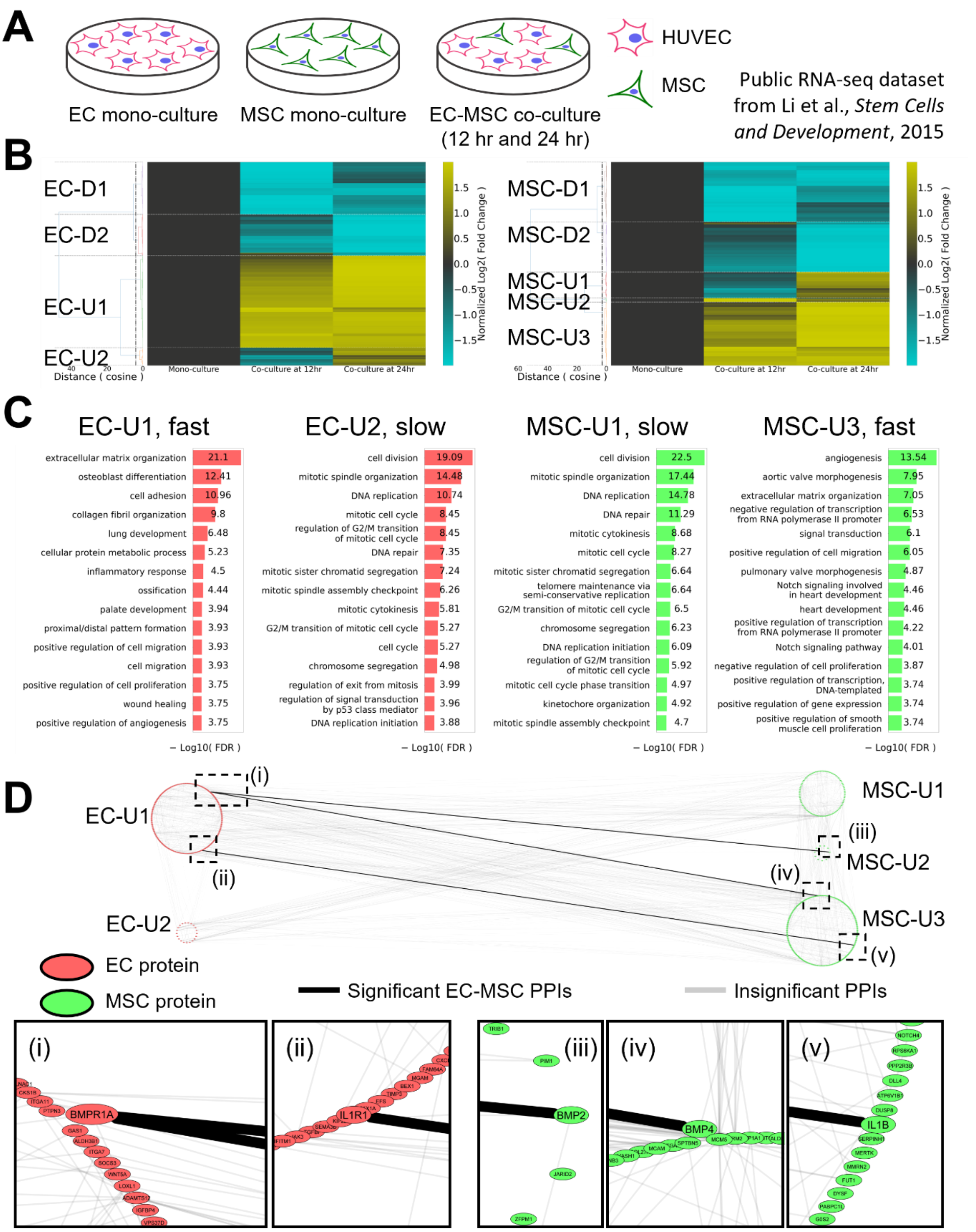
A
EC mono-culture
MSC mono-culture
EC-MSC co-culture
(12 hr and 24 hr)
HUVEC
MSC
Public RNA-seq dataset
from Li et al., *Stem Cells and Development*, 2015
B
EC-D1
EC-D2
EC-U1
EC-U2
Distance (cosine)
Mono-culture
Co-culture at 12hr
Co-culture at 24hr
Normalized Log2(Fold Change)
MSC-D1
MSC-D2
MSC-U1
MSC-U2
MSC-U3
C
EC-U1, fast
extracellular matrix organization 21.1
osteoblast differentiation 12.41
cell adhesion 10.96
collagen fibril organization 9.8
lung development 6.48
cellular protein metabolic process 5.23
inflammatory response 4.5
ossification 4.44
palate development 3.94
proximal/distal pattern formation 3.93
positive regulation of cell migration 3.93
cell migration 3.93
positive regulation of cell proliferation 3.75
wound healing 3.75
positive regulation of angiogenesis 3.75
− Log10(FDR)
EC-U2, slow
cell division 19.09
mitotic spindle organization 14.48
DNA replication 10.74
mitotic cell cycle 8.45
regulation of G2/M transition of mitotic cell cycle 8.45
DNA repair 7.35
mitotic sister chromatid segregation 7.24
mitotic spindle assembly checkpoint 6.26
mitotic cytokinesis 5.81
G2/M transition of mitotic cell cycle 5.27
cell cycle 5.27
chromosome segregation 4.98
regulation of exit from mitosis 3.99
regulation of signal transduction by p53 class mediator 3.96
DNA replication initiation 3.88
− Log10(FDR)
MSC-U1, slow
cell division 22.5
mitotic spindle organization 17.44
DNA replication 14.78
DNA repair 11.29
mitotic cytokinesis 8.68
mitotic cell cycle 8.27
mitotic sister chromatid segregation 6.64
telomere maintenance via semi-conservative replication 6.64
G2/M transition of mitotic cell cycle 6.5
chromosome segregation 6.23
DNA replication initiation 6.09
regulation of G2/M transition of mitotic cell cycle 5.92
mitotic cell cycle phase transition 4.97
kinetochore organization 4.92
mitotic spindle assembly checkpoint 4.7
− Log10(FDR)
MSC-U3, fast
angiogenesis 13.54
aortic valve morphogenesis 7.95
extracellular matrix organization 7.05
negative regulation of transcription from RNA polymerase II promoter 6.53
signal transduction 6.1
positive regulation of cell migration 6.05
pulmonary valve morphogenesis 4.87
Notch signaling involved in heart development 4.46
heart development 4.46
positive regulation of transcription from RNA polymerase II promoter 4.22
Notch signaling pathway 4.01
negative regulation of cell proliferation 3.87
positive regulation of transcription, DNA-templated 3.74
positive regulation of gene expression 3.74
positive regulation of smooth muscle cell proliferation 3.74
− Log10(FDR)
D
EC-U1
EC-U2
MSC-U1
MSC-U2
MSC-U3
(i)
(ii)
(iii)
(iv)
(v)
EC protein
MSC protein
Significant EC-MSC PPIs
Insignificant PPIs
BMPR1A
IL1R1
BMP2
BMP4
IL1B

Figure S6. Identification of key PPIs for EC-MSC crosstalk. A. Experimental design of cell culture of ECs and MSCs from a public dataset of RNA sequencing [1]. **B**. Hierarchical clustering of log2 fold change of gene expression in ECs and MSCs. Expression profiles of ECs and MSCs were classified into 4 and 5 clusters, respectively. Clusters EC-D1, EC-D2, MSC-D1, and MSC-D2 had fast, slow, fast, and slow down-regulation, respectively. Clusters EC-U1, EC-U2, MSC-U1, MSC-U2, and MSC-U3 had fast, slow, slow, fast, and fast up-regulation, respectively. **C**. Gene ontology (GO) analysis of up-regulated gene clusters for ECs and MSCs. Top 15 of significant GO terms for each up-regulated cluster were listed. Negative log-10 values of the false discovery rate (FDR) from the DAVID knowledgebase [2,3] are indicated in bar plots. **D**. Database search of key protein-protein interactions (PPIs). Nodes of EC or MSC proteins were colored in red or, respectively. Edges of significant PPIs between ECs and MSCs were colored in black. Edges of significant PPIs among ECs or MSCs in blue. All the genes in the up-regulated clusters EC-U1, EC-U2, MSC-U1, MSC-U2, and MSC-U3 were entered into a PPI database, STRING (https://string-db.org). Among the PPIs returned, key molecular interactions were screened out with a few scores of confidence in the database. The molecular interactions are visualized using Cytoscape (version 3.9.1).

References.

1. Li J, Ma Y, Teng R, Guan Q, Lang J, Fang J, et al. Transcriptional profiling reveals crosstalk between mesenchymal stem cells and endothelial cells promoting prevascularization by reciprocal mechanisms. Stem Cells Dev. 2015;24:610–23.
2. Huang DW, Sherman BT, Lempicki RA. Systematic and integrative analysis of large gene lists using DAVID bioinformatics resources. Nat Protoc. 2009;4:44–57.
3. Huang DW, Sherman BT, Lempicki RA. Bioinformatics enrichment tools: Paths toward the comprehensive functional analysis of large gene lists. Nucleic Acids Res. 2009;37:1–13.
